# Comprehensive Analysis of the Control of Cancer Stem Cell Characteristics in Endometrial Cancer by Network Analysis

**DOI:** 10.1155/2021/6653295

**Published:** 2021-03-29

**Authors:** Yun Liu, Peigen Chen, Mengxiong Li, Hui Fei, Jinfeng Huang, Tingting Zhao, Tian Li

**Affiliations:** ^1^Department of Obstetrics and Gynecology, The Seventh Affiliated Hospital, Sun Yat-sen University, China; ^2^Reproductive Medicine Center, The Sixth Affiliated Hospital, Sun Yat-sen University, China

## Abstract

**Background:**

Cancer stem cells play an important role in endometrial cancer (EC). It is closely related to self-renewal and therapeutic resistance of EC.

**Methods:**

In this study, WGCNA (weighted gene coexpression network analysis) was used to analyze the relationship between genes and clinical features. We also performed immune cell infiltration analysis of a key module by using ImmuCellAI (Immune Cell Abundance Identifier). Then, key genes were verified in the GEO database. Finally, causal relationship analysis and protein-protein interaction analysis were performed in DisNor tool and STRING.

**Result:**

The mRNA expression-based stemness index (mRNAsi) is significantly lower in normal tissues and is significantly higher in individuals with stage IV or high-grade cancer and those who are obese or postmenopausal. Nineteen key genes (ORC6, C1orf112, RAD54L, SGO2, BUB1, PLK4, KIF18B, BUB1B, TTK, NCAPG, XRCC2, CENPF, KIF15, RACGAP1, ARHGAP11A, TPX2, KIF14, KIF4A, and NCAPH) that were enriched mainly in terms related to the cell cycle and DNA replication were selected by weighted gene coexpression network analysis (WGCNA). Based on the key modules, the numbers of NKT cells, NK cells, and neutrophils in the normal group were significantly higher than those in the cancer group. PLK1, CDK1, and MAD2L1, which were correlated with upstream genes, may be an regulated upstream of key genes.

**Conclusion:**

PLK1, CDK1, and MAD2L1 which were strongly correlated with upstream genes may be a regulated upstream of key genes.

## 1. Introduction

Endometrial cancer (EC) is one of the most common gynecological malignancies. Nearly 382,100 new cases per year are diagnosed worldwide, and 89,900 patients die from the disease each year [1, 2]. Menopause status, diabetes, obesity, the total number of pregnancies, and smoking status are contributing risk factors for EC [3]. The different pathological stages and histological types of this disease have a variable prognosis. Patients with advanced-stage EC are predicted to exhibit poor outcomes. To improve the poor prognoses of people with EC, further investigations of tumorigenic mechanisms are required.

Recent studies have shown that cancer stem-like cells (CSCs) are linked with ECs [4, 5]. CSCs are thought to be crucial in determining recurrence and progression, which are important for cancer metastasis and therapeutic resistance ([5–7]. Cancer stemness has been extensively studied by using deep learning methods. For example, the Progenitor Cell Biology Consortium (https://www.synapse.org/pcbc) has typically used several types of stem cells, such as embryonic stem cells (ESCs), induced pluripotent stem cells (iPSCs), and stem cell- (SC-) derived embryoid bodies (EBs), to define stem cell signatures. A multiplatform analysis that included transcriptomes, methylomes, and transcription factor binding sites was carried out to quantify stemness, resulting in the generation of a DNA methylation-based stemness index (mDNAsi) and a mRNA expression-based stemness index (mRNAsi). Histopathological grades may respond to biological processes in cancer stem cells (CSCs) and tumor dedifferentiation and are indicated by mRNAsi scores. mRNAsi and mDNAsi scores have been calculated for certain CSCs by The Cancer Genome Atlas (TCGA) on the basis of this stemness [8].

Weighted gene coexpression network analysis (WGCNA) is used to construct gene networks in which the correlations between gene sets are identified and weighted by their associated expression levels [9]. WGCNA is a widespread approach for processing gene expression data and investigating network changes. In brief, WGCNA uses topological overlap dissimilarity to estimate the distance between genes and can identify network topologies and subnetworks. Therefore, the gene modules are composed only of highly coexpressed genes that can be associated with clinical features of interest. These modules are tightly correlated with the investigated clinical features and are therefore selected as essential modules for ensuring the accuracy of the results.

Our research investigates the key genes related to stemness by combining WGCNA with the EC mRNAsi in TCGA. The aim of this study is not to develop an innovative method to identify stemness-related genes but rather to obtain new knowledge about the functions of CSC-related genes in cancer.

## 2. Methods

We used R software (version 3.5.1) [10], GraphPad Prism (version 7), and Bioconductor [11] for all statistical analyses in our study.

### 2.1. Data Acquisition and Preprocessing

The RNA sequencing (RNA-seq) data of 587 samples (552 cancer samples and 35 normal samples) were obtained from the University of California Santa Cruz Genome Browser (UCSC: https://xenabrowser.net, version: 2019-07-20), and corresponding clinical data were extracted from the TCGA Pancancer Clinical Data Resource (TCGA-CDR) [12]. According to TCGA-CDR, a progression-free interval (PFI) event was defined as a new tumor event in a patient, including progression of the disease, local recurrence, distant metastasis, new primary tumors at all sites, or death associated with cancer without a new tumor event, including new tumor events whose type was N/A. The mRNA expression-based stemness index (mRNAsi) of the uterine corpus endometrial carcinoma (UCEC) cohort was obtained from a previous study [8]. In total, 563 samples with mRNAsi data were included in our study.

### 2.2. Comparison of mRNAsi among Molecular Subtypes and Different Clinical Features of EC

To investigate the prognostic role of mRNAsi scores in EC, we analyzed the relationship between mRNAsi and clinical features, including histological type, pathological stage, body mass index (BMI), menopause status, and total number of pregnancies, by using GraphPad Prism (version 7).

In addition, the patients were divided into a high-mRNAsi group and a low-mRNAsi group based on the optimal cutpoint chosen by using the “survival” R package [13]. Based on the overall survival (OS) and PFI data obtained from TCGA-CDR, we then performed the log-rank test to compare the OS/PFI between these two groups with a threshold of *p* < 0.05, and Kaplan-Meier curves were also generated.

### 2.3. Selection of Differentially Expressed Genes (DEGs)

DEGs were selected between normal and cancer EC samples by using the “limma” R package [14] with the following criteria: false discovery rate (FDR) < 0.05 and ∣log 2 − fold change | >1. Genes showing a low expression (expression levels < 1) were deleted.

### 2.4. WGCNA

With the “WGCNA” R package [9], WGCNA was performed on selected DEGs showing the highest variance (top 25%). We performed average linkage hierarchical clustering with a minimum gene dendrogram size of 20 by using TOM-based dissimilarity measurements. By analyzing the modules, we calculated the dissimilarity and constructed module dendrograms for these modules.

We then calculated gene significance (GS) to estimate the significance of each module and measure the relationships between genes and sample traits. A cutoff (0.25) was selected to merge the modules based on heights. Next, mRNAsi data and epigenetically regulated mRNAsi data were selected as clinical phenotypes and combined with gene modules for further analysis.

For the identification of key genes, the GS and module membership (MM, the correlation between the genes in the module and their expression profiles) of every key gene were calculated with the following thresholds: cor.gene GS > 0.5 and cor.gene MM > 0.8.

### 2.5. Functional Enrichment Analysis of Key Modules

To understand the biological function of crucial modules selected by WGCNA, Metascape (http://metascape.org), which includes abundant functional annotations, such as KEGG pathway, Reactome pathway, canonical pathway, GO biological process, and CORUM (the comprehensive resource of mammalian protein complexes) annotations, was then used to perform functional enrichment analysis with a *p* value of < 0.001 as the cutoff value [15]. Selected terms with a *p* value of < 0.01 and a number of genes greater than or equal to 3 were considered significant terms.

### 2.6. Immune Cell Infiltration Analysis of Key Modules

Immune Cell Abundance Identifier (ImmuCellAI) is a tool that can calculate the abundance of 18 T cell subsets and 6 other immune cell types (B cells, NK cells, monocytes, macrophages, neutrophils, and DCs) based on RNA-seq data [16]. We used ImmuCellAI to compare the proportions of T cell subsets among key modules.

### 2.7. Validation of the Expression of Key Genes

To verify the expression of key genes in EC, we selected GSE146889, which included 176 samples (91 tumor and 85 normal tissues, 139 MSI and 37 MSS tissues), from the Gene Expression Omnibus (GEO) database to compare the differential expression levels of key genes between the tumor and normal tissues by the Mann–Whitney *U* test. We also compared the MSI and MSS tissues.

### 2.8. Causal Relationship Analysis and Protein-Protein Interaction Analysis

DisNor (https://disnor.uniroma2.it/) is a web-based tool that can generate and explore protein interaction networks based on disease genes by using Mentha protein interaction data and causal interaction information annotated by SIGNOR.

We used DisNor to explore the causal relationships between key genes. Next, STRING (version 11.0, https://string-db.org/) was used to construct a protein-protein interaction network to investigate the interactions among multiple proteins with an interaction score of 0.7 (high confidence).

### 2.9. Gene Coexpression Analysis

To investigate the strength of the relationships between key genes, we then assessed the coexpression relationships between these key genes based on their expression levels through Pearson correlation analysis with the R “corrplot” package.

## 3. Results

### 3.1. Relationships between the mRNAsi and Clinical Features and Molecular Subtypes of EC

**Figure 1:**
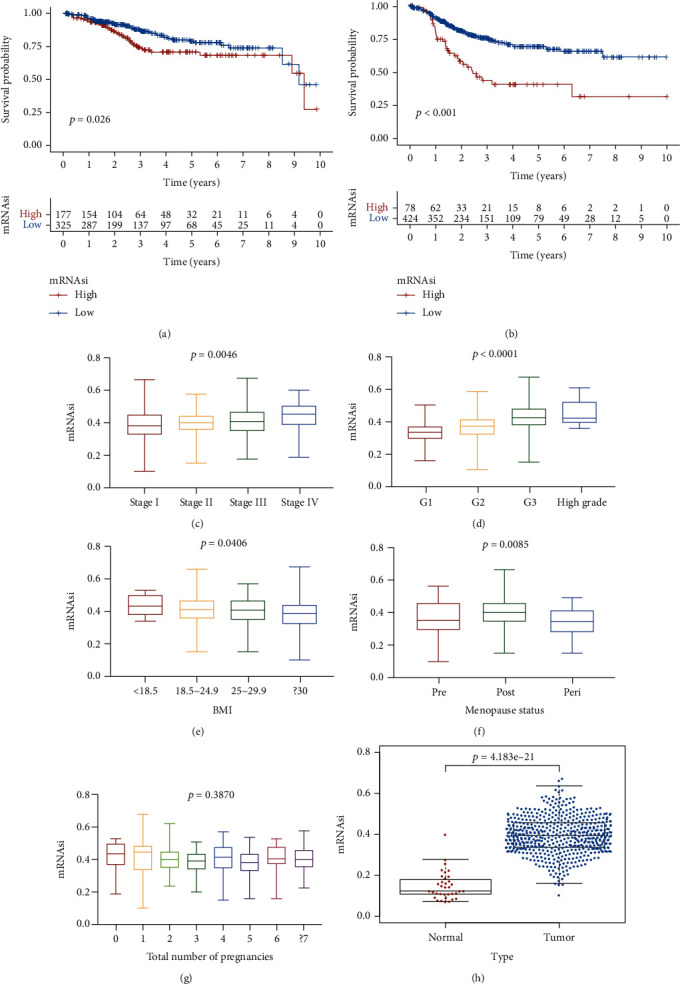
(a) Kaplan-Meier curve of OS time. (b) Kaplan-Meier curve of PFI time. Relationship between mRNAsi and clinical features such as clinical stage (c), pathological grade (d), BMI (e), menopause status (f), and total number of pregnancies (g) (Kruskal-Wallis test). (h) Differences in mRNAsi scores between normal samples and tumor samples.

In total, 563 samples with mRNAsi data were included in our study. As shown in Figure 1(a), the mRNAsi of normal tissues was significantly lower than that of tumor tissues (*p* < 0.0001). In the survival analysis, we found that the OS times (Figure 1(a)) and PFI times (Figure 1(b)) of patients in the low-mRNAsi score group were significantly greater than those of patients in the high-mRNAsi score group.

In the comparison of the mRNAsi among clinical phenotypes of EC, we found that the mRNAsi score was significantly higher in patients with stage IV disease, with an increasing trend from stage I to stage IV (stage I < stage II < stage III < stage IV) (*p* = 0.0046, Kruskal-Wallis test) (Figure 1(c)), and the pathological grade presented the same trend (grade 1 (G1) < grade 2 (G2) < grade 3 (G3) < high-grade) (*p* < 0.0001, Kruskal-Wallis test) (Figure 1(d)). According to the classification system [17] and certain guidelines regarding obesity [18, 19], we then divided the patients into 4 groups based on BMI: underweight (BMI < 18 kg/m^2^), healthy body weight (BMI between 18.5 and 24.9 kg/m^2^), overweight (BMI between 25 and 29.9 kg/m^2^), and obese (BMI > 30 kg/m^2^) (Figure 1(e)). We found a decreasing trend in the mRNAsi score from the underweight to the obese group (*p* = 0.046, one-way ANOVA test). When we compared the mRNAsi scores by menopausal status, we found that the mRNAsi score was significantly higher in patients with postmenopausal status (*p* = 0.0085, Kruskal-Wallis test, Figure 1(f)) (supplementary table 1). When we investigated the relationship between the mRNAsi score and the total number of pregnancies (Figure 1(g)), we found that there was no significant difference in the mRNAsi score among the groups with different numbers of pregnancies.

### 3.2. Selection of DEGs

Selection of DEGs was then performed by using the R “limma” package with the following criteria: false discovery rate (FDR) < 0.05 and ∣log 2 − fold change | >1. After the filtering and normalization of data, differential gene expression analysis was performed. Finally, 3226 DEGs were selected (Figure 1(h)).

### 3.3. WGCNA: Selection of the Most Significant Modules and Genes

**Figure 2:**
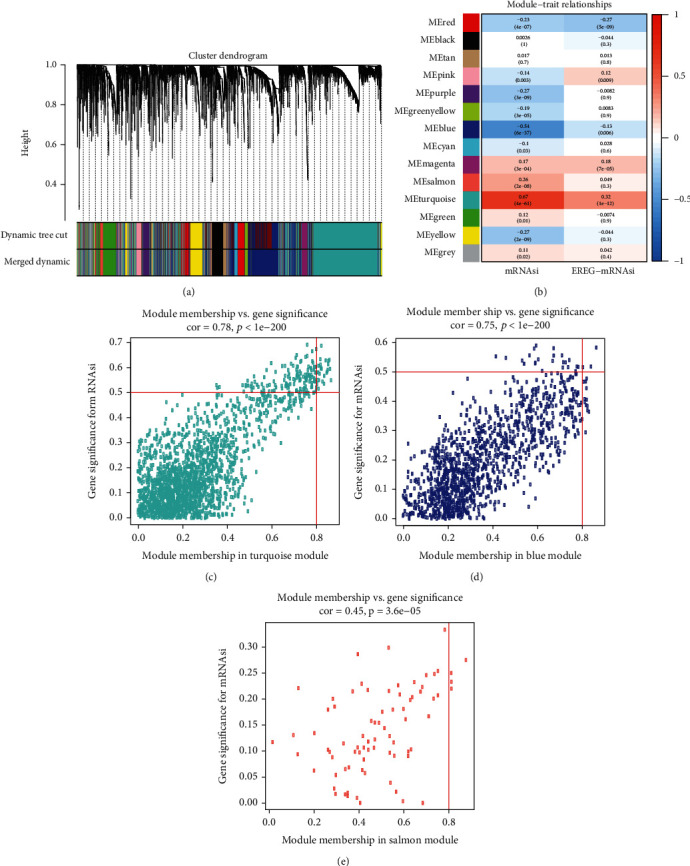
Weighted gene coexpression network for EC. (a) Selected coexpression modules for EC. Each branch corresponds to 11 different modules, and each leaf corresponds to a gene. (b) Relationships between gene modules and mRNAsi or EREG-mRNAsi. The correlation coefficients and corresponding *p* values are also indicated in the figure (inside the brackets is *p* value). Scatter plot of MM and GS in the turquoise (c), blue (d), and salmon (e) modules.

A gene coexpression network was then constructed by WGCNA to select the most significant gene modules and genes. This procedure can also help to elucidate the relationships between genes and EC. We first eliminated the outlier samples (Supplementary Figure 1A), after which 5987 DEGs were selected through cluster analysis, including the 25% of DEGs showing the highest variance in the module, for further analysis. Next, a scale-free network was constructed with a soft threshold of *β* = 4 (SFT.R.sq = 0.930), and 14 modules were selected with a minimum module size of 50 for further analysis (Figure 2(a)).

Then, the overall expression gene level was taken as the MS to estimate the relationships between the corresponding modules and clinical phenotypes (Figure 2(b)). Based on the results, we found that the turquoise module showed the most significant positive correlation with the mRNAsi score (cor = 0.78), and the salmon module also exhibited a positive correlation with the mRNAsi score (cor = 0.45). In addition, the blue module was significantly negatively associated with the mRNAsi score (cor = 0.75) (Figures 2(c)–2(e). Therefore, the turquoise module was chosen for the screening of key genes.

The key gene screening threshold was set as follows: cor.MM > 0.8 and cor.GS > 0.5. Finally, 19 genes were selected: ORC6, C1orf112, RAD54L, SGO2, BUB1, PLK4, KIF18B, BUB1B, TTK, NCAPG, XRCC2, CENPF, KIF15, RACGAP1, ARHGAP11A, TPX2, KIF14, KIF4A, and NCAPH.

### 3.4. Functional Enrichment Analysis of Key Modules

**Figure 3:**
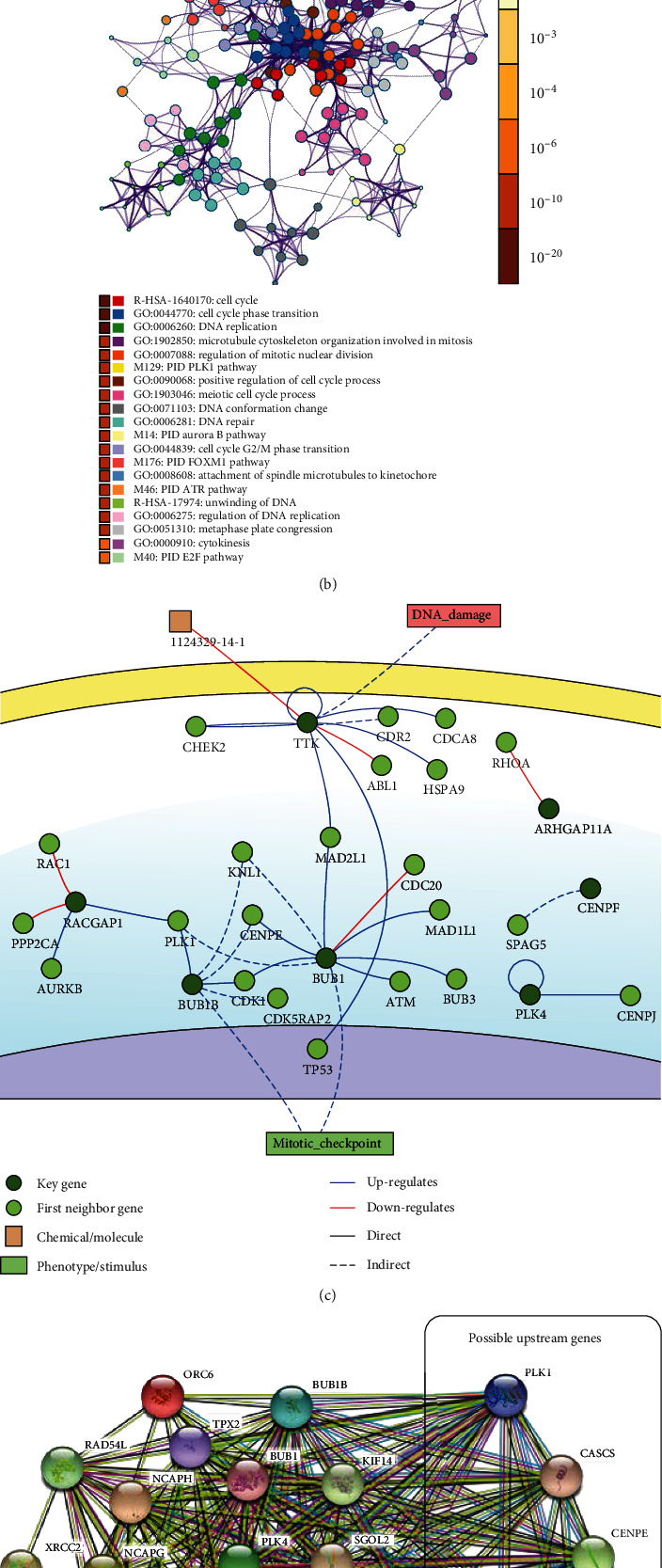
(a) Comparison of the infiltration of immune cells in the turquoise module. (b) Functional enrichment analysis in the turquoise module. (c) Causal interaction analysis of key genes using DisNor. (d) Protein-protein interaction analysis between key genes and upstream genes.

By using Metascape, we found that the genes in the turquoise module were enriched mainly in terms related to the cell cycle (R-HSA-1640170), cell cycle phase transition (GO: 0044770), and DNA replication (GO: 0006260) (Figure 3(b)).

### 3.5. Immune Cell Infiltration Analysis of Key Modules

ImmuCellAI was employed to analyze the infiltration status in the turquoise module. We found that the abundances of naïve CD8 cells (*p* < 0.0001), effector memory cells (*p* < 0.0001), and B cells (*p* < 0.0001) in the cancer group were significantly higher than those in the normal group. We also found that NKT cells (*p* < 0.0001), NK cells (*p* < 0.0001), and neutrophils (*p* < 0.0001) were significantly more abundant in the normal group than in the cancer group (Figure 3(a)).

### 3.6. Comparison and Validation of the Expression Levels of Key Genes

**Figure 4:**
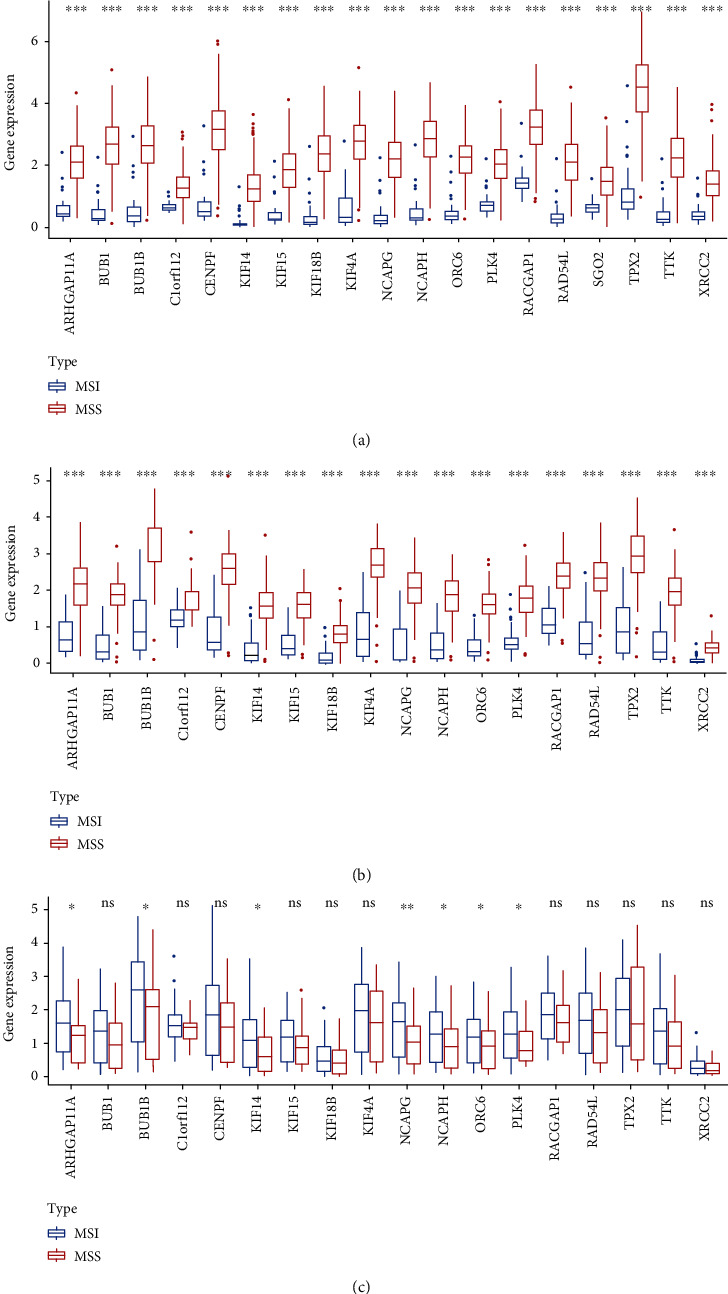
(a) Comparison of the expression levels of key genes between tumor and normal tissues (Mann–Whitney *U* test). Validation of the key genes in the GEO database by using the Mann–Whitney *U* test. (b) Comparison of the expression levels of key genes between the tumor and normal tissues. (c) Comparison of the expression levels of key genes between the MSI and MSS subtypes.

To explore key genes, we then compared the expression levels of these genes between tumor and normal tissues. We found that the expression levels of key genes were significantly higher in tumor tissues (*p* < 0.0001, Figure 4(a)).

Then, we validated the GEO dataset (GSE146889), and the results showed that all 18 genes exhibited significantly higher expression in tumor samples (*p* < 0.0001, Figure 4(b)). We also compared expression levels between the MSI and MSS subtypes. We found that the expression levels of ARHGAP11A, BUB1B, KIF14, NCAPG, NCAPH, ORC6, and PLK4 were significantly higher in the MSI subtype (*p* < 0.05, Figure 4(d)).

### 3.7. Construction of the Interaction and Relationship Network

By using the DisNor tool, we screened the first neighboring genes of the key genes, and we found that some upstream genes, such as PLK1, KNL1 (CASC5), CENPE, CDK1, and MAD2L1, were affected by at least two key genes (Figure 3(c)). KNL1 (CASC5) and CENPE mainly indirectly upregulated key genes. Therefore, PLK1, CDK1, and MAD2L1 were chosen as ideal gene targets for further analysis. Next, to investigate the relationships between the upstream genes and key genes, a protein-protein interaction network was constructed (Figure 3(d)).

### 3.8. Comparison and Validation of the Expression Levels of Key Genes

To explore key genes, we then compared the expression levels of these genes between the tumor and normal tissues. We found that the expression levels of key genes were significantly higher in tumor tissues (*p* < 0.0001, Figure 4(a)).

Then, we validated the GEO dataset (GSE146889), and the results showed that all 18 genes exhibited significantly higher expression in tumor samples (*p* < 0.0001, Figure 4(b)). We also compared expression levels between the MSI and MSS subtypes. We found that the expression levels of ARHGAP11A, BUB1B, KIF14, NCAPG, NCAPH, ORC6, and PLK4 were significantly higher in the MSI subtype (*p* < 0.05, Figure 4(c)).

### 3.9. Coexpression Analysis between Key Genes and Selected Upstream Genes

As shown in Supplement figure 2, we found that the key genes were significantly correlated with the upstream genes PLK1, CDK1, and MAD2L1. The highest correlation was observed between MAD2L1 and SGO2 (*r* = 0.79). We also found that PLK1 was closely related to KIF18B (*r* = 0.71) and that CDK1 was closely related to RACGAP1 (*r* = 0.71), ARHGAP11A (*r* = 0.71), and SGO2 (*r* = 0.71).

## 4. Discussion

EC is one of the most common malignant gynecologic tumors. Recently, an increasing number of studies have shown that CSCs play an essential role in certain features of tumors, such as recurrence, progression, and therapeutic resistance [20]. Therefore, it is essential to screen targets for the treatment of EC stem cells. In this study, we used WGCNA to identify hub genes related to CSC characteristics based on mRNAsi scores. We analyzed the relationships between the mRNAsi scores and the clinical features and molecular subtypes of EC. In the comparison of mRNAsi scores between clinical phenotypes of EC, we found that mRNAsi scores increased as the pathological grade and clinical stage increased, with stage IV and high-grade EC associated with the highest scores.

In contrast, the mRNAsi scores increased as BMI decreased. Moreover, patients with postmenopausal status exhibited the highest mRNAsi scores. When we investigated the relationship between the mRNAsi score and the total number of pregnancies, we found no significant difference in the mRNAsi score among the groups with different numbers of pregnancies. Due to the critical role of pluripotent stem cells in the development of all organ tissues, the key genes may be correlated with the maintenance of stem cell properties in many kinds of cancers. The proteins encoded by these genes are closely related. Therefore, it is necessary to analyze the relationship between CSCs and the progression of EC. It is also crucial to identify genes that may be related to cancer development and progression.

Increasing evidence shows that traditional therapy is not ideal for cancer cells that enter the CSC state by activating the EMT program; thus, the incidence of clinical recurrence mediated by CSC is also high [21]. Additionally, the tumor cells of undifferentiated primary tumors present a greater ability to migrate over long distances, which is more likely to lead to disease progression and a poor prognosis. Cancer progression usually manifests as a loss-of-differentiation phenotype [22]. In this study, the changes in progression after the development of EC were observed to be critical. In the survival analysis, we found that patients with high mRNAsi scores exhibited shorter OS and PFI times. We also noted that stage IV and high-grade EC patients presented increased CSC characteristics, which means that the increase in stem cell properties begins at the initiation stage of metastasis.

The expression level of key genes was higher in the tumor than in normal tissues. When we validated the data in the GEO dataset (GSE146889), we found that the expression levels were significantly related to the tumor subtype. However, we found that the expression levels of ARHGAP11A, BUB1B, KIF14, NCAPG, NCAPH, ORC6, and PLK4 were significantly higher in the MSI subtype, but the other genes did not seem to differ between these two subtypes. These results indirectly indicated that these genes might maintain the characteristics of stem cells.

According to the functional enrichment analysis of the turquoise module, we found that the functional annotations were related mainly to the cell cycle, cell cycle phase transition, and DNA replication. Then, according to the GS and MM, we selected the key genes from the turquoise module. Moreover, we constructed a protein-protein interaction network and observed strong relationships. We found that the key genes were significantly correlated with the upstream genes PLK1, CDK1, and MAD2L1. Based on the protein causality and interaction analysis, PLK1, CDK1, and MAD2L1, all of which affected at least two genes, were selected as ideal treatment targets. Although KNL1 (CASC5) and CENPE also affected two or more genes, their effects are exerted mainly indirectly.

The expression level of polo-like kinase 1 (PLK1), which is the key regulator of mitosis, is increased in various kinds of cancer, such as non-small-cell lung cancer, head and neck cancer, breast cancer, ovarian cancer, EC, and thyroid cancer [23]. Another study showed that PLK1 targeting combined with low-dose cisplatin therapy exhibited strong antitumor efficacy by inducing arrest at the G2/M checkpoint, increasing DNA breakage, and sensitizing tumor cells to antitumor drugs [24]. Cyclin-dependent kinase 1 (CDK1) plays an important role in the regulation of PLK1 activity at the G2/M transition in EC [25]. Studies have shown that mitotic arrest deficient 2-like 1 (MAD2L1) is closely related to lymph node metastasis in EC [26]. Therefore, we speculate that these genes affect tumor biological behavior by affecting the characteristics of CSCs in EC.

In conclusion, 19 key genes identified in this study play critical roles in the biological behavior of EC stem cells. Three upstream genes, PLK1, CDK1, and MAD2L1, may be potential targets for EC treatment by inhibiting the characteristics of EC stem cells. However, the results of this study were based on public data; thus, additional biological studies are needed to further validate these findings.

## Data Availability

The data that support the findings of this study are available the University of California Santa Cruz Genome Browser (UCSC: https://xenabrowser.net, version: 2019-07-20), and corresponding clinical data were extracted from the TCGA Pancancer Clinical Data Resource (TCGA-CDR). The validation dataset was obtained from the Gene Expression Omnibus (GEO) database (GSE146889).

## Abbreviations

EC: Endometrial cancer
WGCNA: Weighted gene coexpression network analysis
mRNAsi: mRNA expression-based stemness index
CSCs: Cancer stem-like cells
TCGA-CDR: TCGA Pancancer Clinical Data Resource
OS: Overall survival
PFI: Progression-free interval
FDR: False discovery rate
GS: Gene significance.

## Supplementary Materials

Supplementary 1 Supplementary Figure 1: sample clustering to detect outliers, clustering of module eigengenes, and the trait heat map of the weighted gene coexpression network of endometrial cancer.

Supplementary 2 Supplementary Figure 2: correlation analysis between key genes and PLK1, MAD2L1, and CDK1.

Supplementary 3 Table 1: the data source of Figure 1.
