# Supplementary figures and images for: Comprehensive Analysis of the Control of Cancer Stem Cell Characteristics in Endometrial Cancer by Network Analysis
Source: Comput Math Methods Med. 2021 Mar 29;2021:6653295. doi: 10.1155/2021/6653295 (PMC8025127; doi:10.1155/2021/6653295)

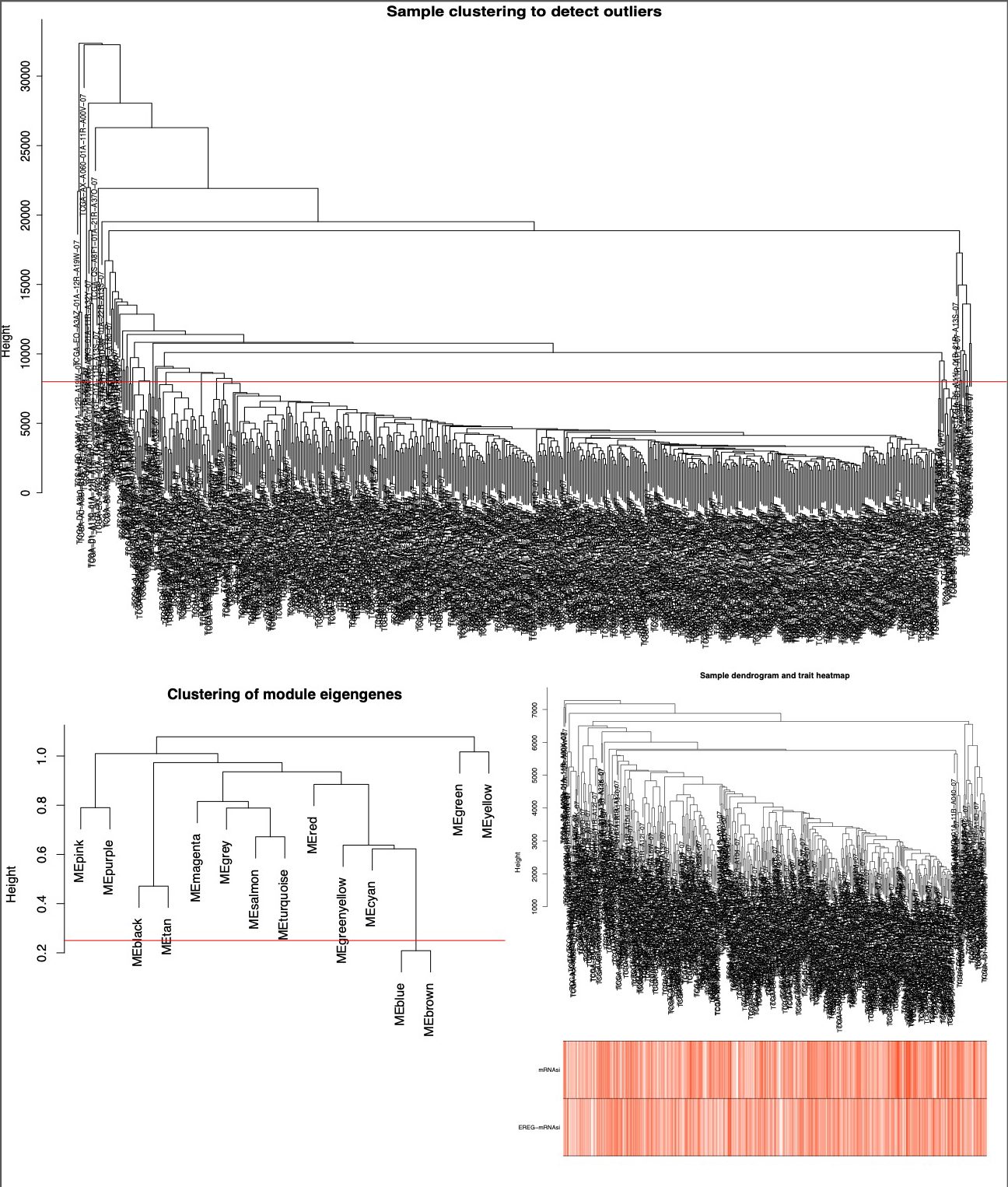

Supplement: Supplementary 1 — Supplementary Figure 1: sample clustering to detect outliers, clustering of module eigengenes, and the trait heat map of the weighted gene coexpression network of endometrial cancer. [file 6653295.f1.jpeg]

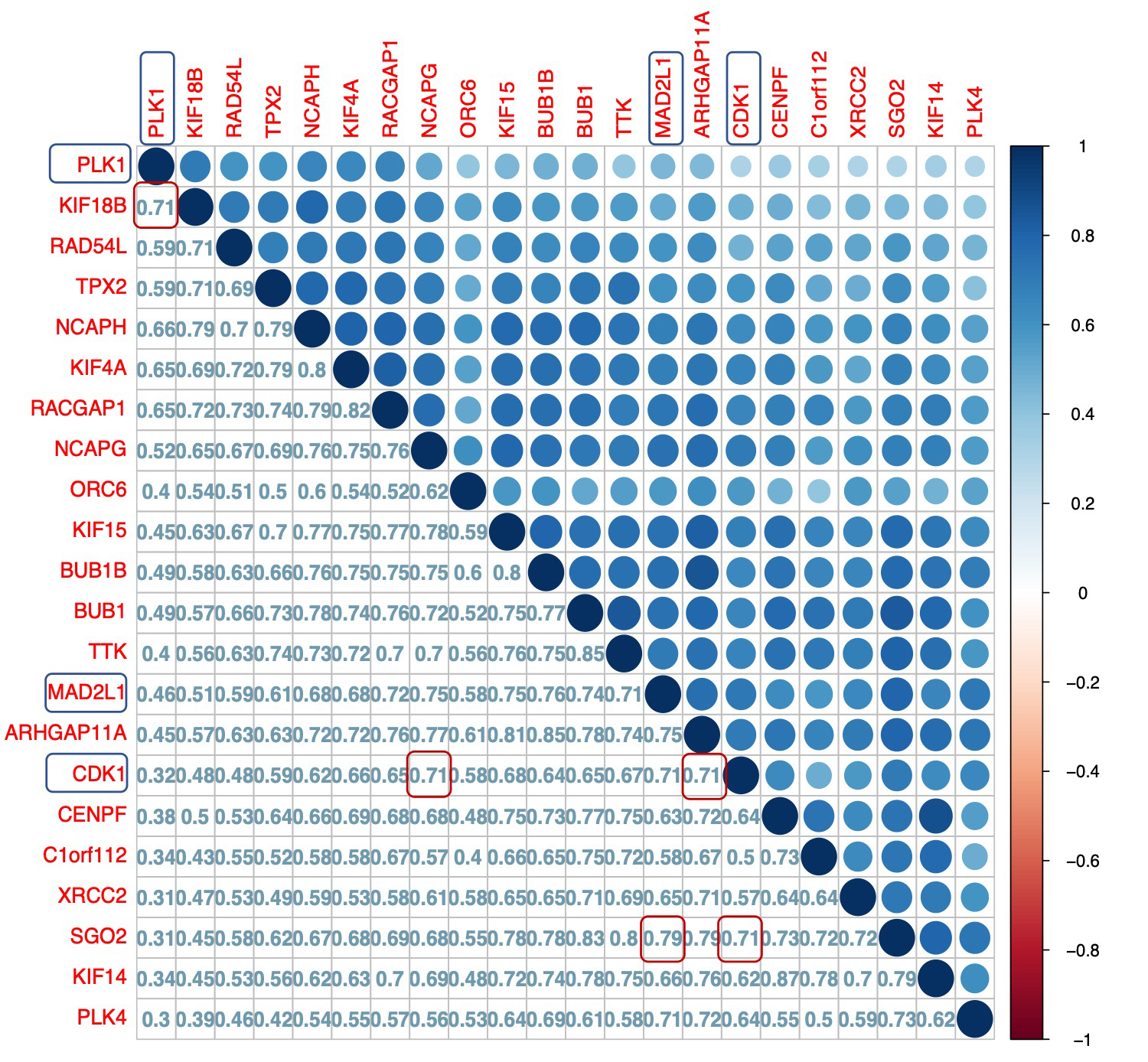

Supplement: Supplementary 2 — Supplementary Figure 2: correlation analysis between key genes and PLK1, MAD2L1, and CDK1. [file 6653295.f2.jpeg]
