# Supplementary material for: Comprehensive Analysis of the Control of Cancer Stem Cell Characteristics in Endometrial Cancer by Network Analysis
Source: Comput Math Methods Med. 2021 Mar 29;2021:6653295. doi: 10.1155/2021/6653295 (PMC8025127; doi:10.1155/2021/6653295)
Supplement: Supplementary 3 — Table 1: the data source of Figure 1. [file 6653295.f3.docx]

| Table Analyzed | Stage | Grade |
| --- | --- | --- |
|  |  |  |
| Kruskal-Wallis test |  |  |
| P value | 0.0021 | <0,0001 |
| Exact or approximate P value? | Approximate | Approximate |
| P value summary | ** | **** |
| Do the medians vary signif. (P < 0.05)? | Yes | Yes |
| Number of groups | 4 | 4 |
| Kruskal-Wallis statistic | 14.69 | 108.7 |
|  |  |  |
| Data summary |  |  |
| Number of treatments (columns) | 4 | 4 |
| Number of values (total) | 528 | 528 |

| BMI |  |  |  |  |  |
| --- | --- | --- | --- | --- | --- |
| ANOVA table | SS | DF | MS | F (DFn, DFd) | P value |
| Treatment (between columns) | 0.06529 | 3 | 0.02176 | F (3, 493) = 2,779 | P=0,0406 |
| Residual (within columns) | 3.861 | 493 | 0.007831 |  |  |

| total_number_of_pregnancies |  |  |  |  |  |
| --- | --- | --- | --- | --- | --- |
| ANOVA table | SS | DF | MS | F (DFn, DFd) | P value |
| Treatment (between columns) | 0.046 | 7 | 0.006571 | F (7, 247) = 0,8666 | P=0,5335 |
| Residual (within columns) | 1.873 | 247 | 0.007582 |  |  |
| Total | 1.919 | 254 |  |  |  |
